# The Development and Characterization of Near-Isogenic and Pyramided Lines Carrying Resistance Genes to Brown Planthopper with the Genetic Background of *Japonica* Rice (*Oryza sativa* L.)

**DOI:** 10.3390/plants8110498

**Published:** 2019-11-12

**Authors:** Cuong D. Nguyen, Holden Verdeprado, Demeter Zita, Sachiyo Sanada-Morimura, Masaya Matsumura, Parminder S. Virk, Darshan S. Brar, Finbarr G. Horgan, Hideshi Yasui, Daisuke Fujita

**Affiliations:** 1 United Graduate School of Agricultural Sciences, Kagoshima University, Kagoshima 890-0065, Japan; cuongdhqt2000@gmail.com; 2 College of Food Industry, 101B Le Huu Trac Street, Son Tra District, Da Nang City 550000, Vietnam; 3 International Rice Research Institute, DAPO Box 7777, Metro Manila 1301, Philippines; h.verdeprado@irri.org (H.V.); p.virk@cgiar.org (P.S.V.); darshanbrar@pau.edu (D.S.B.); 4 Faculty of Agriculture, Saga University, Saga 840-8502, Japan; demeterzita2012@gmail.com; 5 NARO Kyushu Okinawa Agricultural Research Center, 2421 Suya, Koshi, Kumamoto 861–1192, Japan; sanadas@affrc.go.jp (S.S.-M.); mmasa@affrc.go.jp (M.M.); 6 International Center for Tropical Agriculture, A.A, 6713 Cali, Colombia; 7 School of Agricultural Biotechnology, Punjab Agricultural University, Ludhiana 141027, India; 8 EcoLaVerna Integral Restoration Ecology, Bridestown, Kildinan, Co. Cork, T56 CD39, Ireland; f.g.horgan@gmail.com; 9 Plant Breeding Laboratory, Graduate School, Kyushu University, Fukuoka 812-8581, Japan; hyasui@agr.kyushu-u.ac.jp

**Keywords:** rice (*Oryza sativa* L.), brown planthopper, near-isogenic lines, pyramided lines, resistance, virulence

## Abstract

The brown planthopper (BPH: *Nilaparvata lugens* Stål.) is a major pest of rice, *Oryza sativa*, in Asia. Host plant resistance has tremendous potential to reduce the damage caused to rice by the planthopper. However, the effectiveness of resistance genes varies spatially and temporally according to BPH virulence. Understanding patterns in BPH virulence against resistance genes is necessary to efficiently and sustainably deploy resistant rice varieties. To survey BPH virulence patterns, seven near-isogenic lines (NILs), each with a single BPH resistance gene (*BPH2*-NIL, *BPH3*-NIL, *BPH17*-NIL, *BPH20*-NIL, *BPH21*-NIL, *BPH32*-NIL and *BPH17-ptb*-NIL) and fifteen pyramided lines (PYLs) carrying multiple resistance genes were developed with the genetic background of the *japonica* rice variety, Taichung 65 (T65), and assessed for resistance levels against two BPH populations (Hadano-66 and Koshi-2013 collected in Japan in 1966 and 2013, respectively). Many of the NILs and PYLs were resistant against the Hadano-66 population but were less effective against the Koshi-2013 population. Among PYLs, *BPH20+BPH32*-PYL and *BPH2+BPH3+BPH17*-PYL granted relatively high BPH resistance against Koshi-2013. The NILs and PYLs developed in this research will be useful to monitor BPH virulence prior to deploying resistant rice varieties and improve rice’s resistance to BPH in the context of regionally increasing levels of virulence.

## 1. Introduction

The brown planthopper (BPH: *Nilaparvata lugens* Stål.) is a major pest of rice (*Oryza sativa* L.) in tropical and subtropical Asia [1]. BPH damages rice by sucking phloem from the plants (mechanical damage) or by transmitting viruses such as rice grassy stunt virus (RGSV), rice ragged stunt phytoreovirus (RRSV) and rice wilted stunt virus (RWSV) [2,3]. At high planthopper densities, rice crops display patches of desiccated rice known as ‘hopperburn.’ Insecticides have been widely used to reduce BPH populations [4]. However, insecticides are damaging to human health and the environment, and are increasingly recognized as contributing to BPH outbreaks through physiological and ecological pest resurgence mechanisms [1]. Host plant resistance is considered a potentially effective alternative to harmful insecticides, that reduces BPH damage without detrimental effects on the natural enemies of BPH [5].

To date, more than 34 BPH resistance genes have been identified from rice cultivars and wild rice species. Seven genes: *BPH9*, *BPH14*, *BPH17*, *BPH18*, *BPH26*, *BPH29* and *BPH32* have been cloned and characterized for different BPH resistance levels [6,7,8,9,10,11,12]. Four gene clusters (chromosomal regions) strongly associated with BPH resistance have been identified. These occur on chromosomes 4S (short arm), 4L (long arm), 6S and 12 [3,13]. Four genes (*BPH12* from B14, *BPH15*, *BPH17* and *BPH20*) and six quantitative trait loci (QTLs) (*QBph4, QBPH4.1, QBPH4.2, QBph4.2 qBph4.3* and *qBph4.4*) for BPH resistance have been identified on chromosome 4S [13,14,15,16,17,18,19,20]. Among those QTLs, *QBph4* (6.7–6.9 Mbp) from IR02W101 (*Oryza officinalis*) and *QBph4*.2 (6.6–6.9 Mbp) from IR65482-17 (*Oryza australiensis*) were identified at similar locations based on physical distance; *QBPH4.1* (5.8–7.8 Mbp) and *QBPH4.2* (15.2–17.2 Mbp) were identified from Rathu Heenati; *qBph4.3* (0.2–0.7 Mbp) and *qBph4.4* (0.7–13.1 Mbp) were detected in Salkathi. On chromosome 4L, five genes for BPH resistance—*BPH6*, *BPH12(t)* from GSK185-2, *BPH18(t)* from BPH2183, *BPH27* from GX2183 and *BPH27(t)* from Balamawee have been identified [21,22,23,24,25]. Six genes/QTLs have been detected on the short arm of chromosome 6: *BPH3*, *BPH4*, *BPH25*, *BPH32, Qbph6* and *qBPH6(t)* [12,26,27,28,29,30]. Eight genes have been identified on the long arm of chromosome 12—*BPH1*, *BPH2*, *BPH7*, *BPH9*, *BPH10* and *BPH18* from IR65482-7-216-1-2, and *BPH21* and *BPH26* [17,29,31,32,33,34,35,36,37,38]. The BPH resistance genes on chromosome 12 were classified into four allelic types based on their amino acid sequences and different resistance levels: type 1—*BPH1, BPH10, BPH18* and *BPH21*; type 2—*BPH2* and *BPH26*; type 3—*BPH7*; and type 4—*BPH9* [6]. A number of highly resistant rice breeding lines and varieties contain several genes with resistance to BPH and other phloem feeding Hemiptera. These include IR71033-121-15 introgressed from *Oryza minuta* carrying *BPH20*, *BPH21* and *qBPH6(t)* [17,39]; Rathu Heenati carrying *BPH3*, *BPH17*, *QBPH4.1* and *QBPH4.2* [8,13,27]; and PTB33 carrying *BPH2* and *BPH32* [12,40,41].

Monogenic resistance is vulnerable to rapid adaptation by BPH populations. Research indicates that BPH populations have sufficient genetic variability to enable them to overcome specific resistance genes when selected on a resistant host over multiple generations [4,42,43]. In the late 1970s, BPH populations adapted to varieties carrying the *BPH1* and/or *BPH2* genes after these were widely deployed in rice varieties across Asia [4,42,44]. A recent multi-national study has indicated that BPH populations across much of Asia have adapted to feed on rice carrying the *BPH1, BPH2, BPH5, BPH7, BPH8, BPH9, BPH10* and *BPH18* genes [45]. Under laboratory conditions, BPH populations continually reared for between seven to 15 generations on resistant rice varieties were capable of adapting to resistance from a range of genes, including *BPH1, BPH2, BPH3, BPH8, BPH9, BPH10* and *BPH32* [43,46,47,48,49]. Through adaptation to resistance genes, BPH acquires stronger virulence against resistance genes and BPH virulence remains stable for several decades [4,50]. Therefore, it is important to preserve the effects of resistance genes by preventing BPH adaptation.

To prevent further adaptation by BPH populations to available resistance genes, a strategy for deploying resistance based on insect virulence is necessary [4]. However, BPH virulence varies under different environments depending on the predominant rice cultivars, BPH migration routes, and the length of population exposure to different resistance genes [4]. Without exposing resistance genes to BPH populations under controlled conditions prior to deployment, the potential effectiveness of the resistance genes for target regions is difficult to predict. In previous studies, the virulence of BPH has been characterized using resistant varieties [45,48,51,52,53]. However, many BPH-resistant varieties have multiple resistance genes, such that the effects of any single resistance gene cannot be assessed using these varieties. In contrast, the effects of any single resistance gene may be revealed in detail by using near-isogenic lines (NILs) that carry the gene on the genetic background of a susceptible variety. Recently, more than 16 NILs with BPH resistance genes (*BPH3*, *BPH4*, *BPH6*, *BPH9*, *BPH10*, *BPH12*, *BPH14*, *BPH15*, *BPH17*, *BPH18*, *BPH20*, *BPH21*, *BPH25*, *BPH26, BPH30* and *BPH32*) have been developed on the genetic backgrounds of several *indica* and *japonica*-susceptible varieties. These NILs have been evaluated against different BPH populations from China, the Philippines and Japan [14,54,55,56,57,58].

Because the resistance of rice varieties carrying single genes is weaker and less durable (i.e., allowing rapid BPH adaptation) to BPH than varieties with multiple resistance genes, several researchers have proposed the pyramiding of two or more genes to enhance resistance levels and thereby avoid pest adaptation [59]. Combinations of multiple BPH resistance genes have been reported to increase levels of plant resistance to BPH. For example, a pyramided line (PYL) with *BPH14* and *BPH15* enhanced resistance against BPH from China compared to monogenic NILs with either *BPH14* or *BPH15* alone [60]. Similarly, the pyramided lines *BPH6* + *BPH12* PYL and *BPH3 + BPH27* PYL exhibited greater resistance levels in bulk seedling tests than monogenic lines with each of the genes present alone [14,55], and *BPH17 + BPH21* PYL had greater resistance against BPH in the Philippines than lines with either gene alone [57]. Pyramiding the *BPH25* and *BPH26* genes into a single rice line was reported to have positive epistatic effects against BPH populations collected in Vietnam, the Philippines and Japan [51,61]. Therefore, the development of rice varieties carrying multiple BPH resistance genes might be an effective way to enhance BPH resistance. 

In this study, seven NILs with BPH resistance genes (*BPH2*, *BPH3*, *BPH17, BPH20*, *BPH21, BPH32* and *BPH17-ptb*) and a *japonica* rice genetic background were developed to evaluate the effects of different resistance genes on BPH populations. Based on the NILs developed, 15 pyramided lines (PYLs) carrying two or three resistance genes were developed to enhance levels of resistance against BPH. Additionally, using the NILs and PYLs we developed, the study compared resistance against two BPH populations collected in Japan: the first was collected in 1966 (before resistant varieties were widely released) and the second was collected in 2013 (recently migrated from China to Japan). Comparisons of the reactions by BPH from each population to the NILs and PYLs indicates the utility of resistance genes and their different combinations (some with epistatic effects) against modern BPH populations. 

## 2. Results

### 2.1. Development of Seven NILs for BPH Resistance

**Figure 1:**
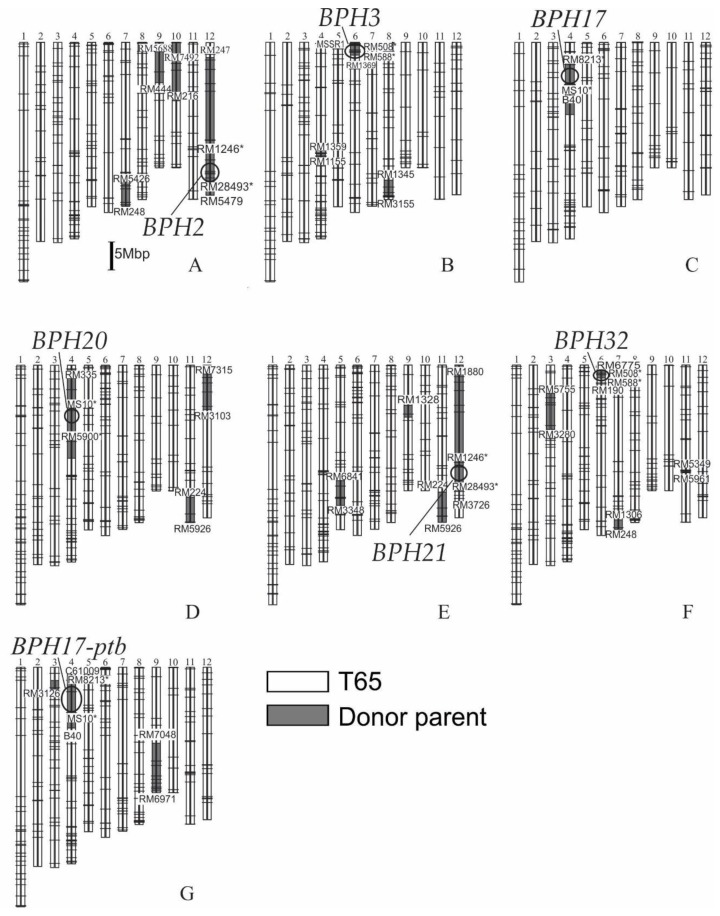
Graphical genotypes of *BPH2*-NIL (**A**), *BPH3*-NIL (**B**), *BPH17*-NIL (**C**), *BPH20*-NIL (**D**), *BPH21*-NIL (**E**), *BPH32*-NIL (**F**) and *BPH17-ptb*-NIL (**G**). The 12 bars indicate 12 chromosomes of rice. Horizontal lines across the chromosomes show the positions of polymorphic SSR markers. Circles indicate the approximate positions of brown planthopper resistant genes. The asterisks (*) indicate SSR markers that were used for marker-assisted selection.

Seven NILs with BPH resistance genes from three donor parents on the genetic background of Taichung 65 (T65) were developed through marker-assisted selection (MAS) and backcrossing (Table 1 and Table 2). For three donor parents, IR71033-121-15 has *BPH20* and *BPH21*; Rathu Heenati contains *BPH3* and *BPH17*; and PTB33 carries *BPH2, BPH17-ptb* and *BPH32* based on previous studies. For PTB33, there has been no previous report of a BPH resistance gene on chromosome 4S. However, amino acid sequences for the *BPH17* locus in PTB33 were identical to those of Rathu Heenati [8]. Thus, we assume that PTB33 contains a gene for BPH resistance on chromosome 4S and tentatively named this as *BPH17-ptb*. The substituted chromosomal segments of the NILs were detected by polymorphic simple sequence repeat (SSR) markers that were equally distributed across the whole genome (Table 3; Figure 1). The genetic background of *BPH2*-NIL was analyzed using 203 polymorphic SSR markers. The ratio of substituted segments from PTB33 on *BPH2*-NIL was 9.1–14.8% (total 33.9–55.0 Mbp). One substituted segment with a size of 21.3–25.4 Mbp encompassing *BPH2* was detected between RM247 and RM5479 on chromosome 12. The other three segments were detected between RM5426 and RM248 on chromosome 7 with a size of 3.4–4.2 Mbp, between RM5688 and RM444 on chromosome 9 with a size of 4.2–9.2 Mbp and between RM7492 and RM216 on chromosome 10 with a size of 5.0–16.2 Mbp. 

The genetic background of *BPH3*-NIL was confirmed using 195 polymorphic SSR markers, and the ratio of substituted segment from Rathu Heenati was 1.0–3.0% (total 3.8–11.3 Mbp). One segment with a size of 1.6–1.8 Mbp including *BPH3* was detected between MSSR1 and RM1369 on the short arm of chromosome 6. The other substituted segments were detected between RM1359 and RM1155 on chromosome 4 with a size of 0.5–4.2 Mbp and between RM1345 and RM3155 on chromosome 8 with a size of 1.8–4.9 Mbp. 

The genetic background of *BPH17*-NIL was surveyed using 173 polymorphic SSR markers. The ratio of substituted segments was 1.0–4.8% (total 3.8–17.6 Mbp) containing one segment located between RM8213 and B40 on chromosome 4, including the *BPH17* region. 

The genetic background of *BPH17-ptb*-NIL was analyzed using 229 polymorphic SSR markers, and the ratio of substituted segments from PTB33 on *BPH17-ptb*-NIL was 2.8–7.6% (total 10.5–28.1 Mbp). One substituted segment with 5.8–13.1 Mbp encompassing *BPH17-ptb* was detected between C61009 and B40 on chromosome 4. Two other substituted segments were detected at RM3126 on chromosome 3 and between RM7048 and RM6971 on chromosome 9 (4.7–11.5 Mbp). 

The genetic background of *BPH20*-NIL was confirmed using 237 polymorphic SSR markers and the ratio of substituted segments of IR71033-121-15 was 5.6–9.6% (total 20.6–35.5 Mbp). One segment with a size of 13.8–19.9 Mb containing *BPH20* was detected between RM335 and RM5900 on chromosome 4. Two other substituted segments were detected between RM224 and RM5926 on chromosome 11 (1.5–7.7 Mbp) and between RM7315 and RM3103 on chromosome 12 (5.3–7.9 Mbp). 

The genetic background of *BPH21*-NIL was surveyed using 229 polymorphic SSR markers, and the ratio of substituted segments from IR71033-121-15 was 7.1–11.6% (total 26.4–43.1 Mbp). One segment with a size of 22.6–23.7 Mbp, including *BPH21*, was detected between RM1880 and RM28493 on chromosome 12. Three other segments were detected between RM6841 and RM3348 on chromosome 5 (2.3–7.2 Mbp), around RM1328 on chromosome 9 and between RM224 and RM5926 on chromosome 11 (1.5–7.7 Mbp). 

The genetic background of *BPH32*-NIL was confirmed using 233 polymorphic SSR markers. The ratio of substituted segments of PTB33 on *BPH32*-NIL was 1.9–4.1% (total 7.1–15.1 Mbp). One segment with a size of 1.6–3.2 Mbp containing *BPH32* was detected between RM6775 and RM190 on chromosome 6. Three other segments from the donor parent were detected between RM5755 and RM3280 on chromosome 3 with a size of 4.9–8.1 Mbp, between RM1306 and RM248 on chromosome 7 with a size of 0.4–3.2 Mbp and between RM5349 and RM5961 on chromosome 11 with a size of 0.2–0.6 Mbp. 

### 2.2. Development of 15 PYLs Carrying Two or Three BPH Resistance Genes

Twelve PYLs carrying two BPH resistance genes (BPH2 + BPH17-PYL, BPH2 + BPH25-PYL, BPH2 + BPH32-PYL, BPH2 + BPH17-ptb-PYL, BPH3 + BPH17-PYL, BPH17 + BPH21-PYL, BPH20 + BPH21-PYL, BPH20 + BPH32-PYL, BPH21 + BPH25-PYL, BPH21 + BPH17-ptb-PYL, BPH25 + BPH17-ptb-PYL and BPH32 + BPH17-ptb-PYL) and three PYLs containing three BPH resistance genes (BPH2 + BPH3 + BPH17-PYL, BPH2 + BPH32 + BPH17-ptb-PYL and BPH20 + BPH21 + BPH32-PYL) were developed using NILs and PYLs with BPH resistance gene(s) (Table 2). The PYLs were confirmed for resistance genes through foreground selection using flanking SSR markers tightly linked to each resistance gene. Most of PYLs were selected from the BC_4_F_3_ equivalent generation, except BPH3 + BPH17-PYL from the BC_4_F_4_ equivalent generation, BPH20+BPH21-PYL from the BC_3_F_8_ generation and BPH32 + BPH17-ptb-PYL from the BC_3_F_8_ generation.

### 2.3. Comparison of Resistance Levels against Hadano-66 by Modified Seedbox Screening Test ( MSST)

**Figure 2:**
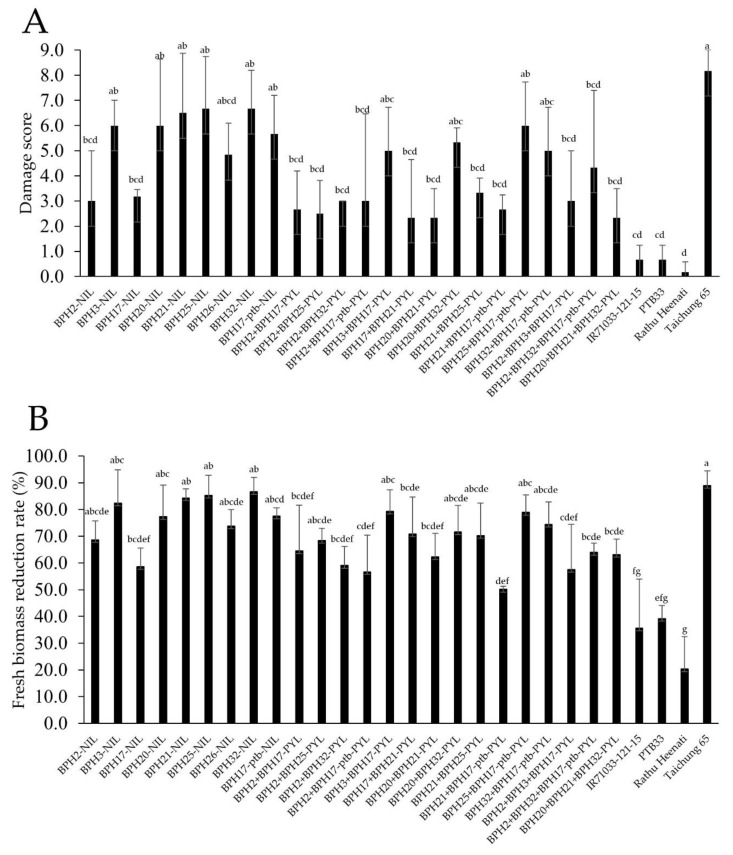
Damage scores (**A**) and fresh biomass reduction rates (**B**) of near-isogenic lines and pyramided lines infested with the Hadano-1966 *Nilaparvata lugens* population using the modified seedbox screening test at the seedling stage. The lower damage scores and fresh biomass reduction rates indicate higher resistance levels.

T65 was highly damaged (damage score (DS) = 8.2) by the Hadano-66 population (Figure 2A). The DSs of the donor parents were significantly lower (0.7 for IR71033-121-15, 0.7 for PTB33 and 0.2 for Rathu Heenati) than that of T65. The donor parents also had higher levels of resistance compared with their respective NILs and PYLs. Among the NILs, *BPH2*-NIL (DS: 3.0) and *BPH17*-NIL (3.2) showed the highest resistance levels. The other NILs *BPH3*-NIL (6.0), *BPH20*-NIL (6.0), *BPH21*-NIL (6.5), *BPH25*-NIL (6.7), *BPH26*-NIL (4.8), *BPH32*-NIL (6.7) and *BPH17-ptb*-NIL (5.7), had lower DSs than T65’s but were not significantly different from the T65. Damage scores across the 15 PYLs ranged from 2.3 to 6.0. Among PYLs, the DSs of 10 PYLs—*BPH2 + BPH17*-PYL (2.7), *BPH2 + BPH25*-PYL (2.5), *BPH2 + BPH32*-PYL (3.0), *BPH2 + BPH17-ptb*-PYL (3.0), *BPH17 + BPH21*-PYL (2.3), *BPH20 + BPH21*-PYL (2.3), *BPH21 + BPH25*-PYL (3.3), *BPH21 + BPH17-ptb*-PYL (2.7), *BPH2 + BPH3 + BPH17*-PYL (3.0) and *BPH20 + BPH21 + BPH32*-PYL (2.3), were equal to or less than 3.3, while the DSs of five PYLs—*BPH3 + BPH17*-PYL (5.0), *BPH20 + BPH32*-PYL (5.3), *BPH25 + BPH17-ptb*-PYL (6.0), *BPH32 + BPH17-ptb* (5.0) and *BPH2 + BPH32 + BPH17-ptb*-PYL (4.3), were more than 4.3. Although the DSs between NILs and PYLs were not significantly different, the resistance levels of the PYLs tended to be higher than those of the NILs.

Additionally, fresh biomass reduction rates (FBRRs) of the NILs and PYLs were calculated as an indicator of resistance (Figure 2B). T65 had the highest FBRR (89.0%) and was significantly different from the donor parents: IR71033-121-15 (35.7%), PTB33 (39.2%) and Rathu Heenati (20.4%). Among the NILs, *BPH17*-NIL (58.7%) had the lowest FBRR and was significantly different from T65. The other NILs, *BPH2*-NIL (68.6%), *BPH3*-NIL (82.4%), *BPH20*-NIL (77.3%), *BPH21*-NIL (84.3%), *BPH25*-NIL (85.3%), *BPH26*-NIL (73.8%), *BPH32*-NIL (86.7%) and *BPH17-ptb*-NIL (77.6%), had lower FBRRs than T65; however, the differences were not significant. The FBRRs of four PYLs—*BPH2 + BPH32*-PYL (59.1%), *BPH2* + *BPH17-ptb*-PYL (56.7%), *BPH21* + *BPH17-ptb*-PYL (50.1%) and *BPH2* + *BPH3* + *BPH17*-PYL (57.6%), were less than 60%. The FBRRs of five PYLs—*BPH2* + *BPH17*-PYL (64.5%), *BPH2* + *BPH25*-PYL (68.4%), *BPH20* + *BPH21*-PYL (62.3%), *BPH2* + *BPH32* + *BPH17-ptb*-PYL (64.0%) and *BPH20* + *BPH21* + *BPH32*-PYL (63.2%), ranged from 60% to 70%; and the FBRRs of six PYLs—*BPH3* + *BPH17*-PYL (79.3%), *BPH17* + *BPH21*-PYL (70.9%), *BPH20* + *BPH32*-PYL (71.6%), *BPH21* + *BPH25*-PYL (70.3%), *BPH25* + *BPH17-ptb*-PYL (78.9%) and *BPH32* + *BPH17-ptb*-PYL (74.4%), ranged from 70% to 80%. Additionally, DSs and FBRRs were positively correlated (Pearson’s C = 0.89; *p* < 0.001). 

### 2.4. Comparison of Adult Mortality (ADM) of the Hadano-66 Population to the NILs and PYLs

Levels of adult mortality (ADM) of the donor parents IR71033-121-15, PTB33 and Rathu Heenati (100%) were significantly higher than that of T65 (17.6%) (Table 4). Among the NILs, *BPH2*-NIL and *BPH17*-NIL had the highest ADM rates, 68.9% and 59.0%, respectively. The ADM rates of other NILs were not significantly different from that of T65. The PYLs carrying the *BPH2—BPH2 + BPH17*-PYL (75.0%), *BPH2 + BPH25*-PYL (87.5%), *BPH2 + BPH32*-PYL (84.0%) and *BPH2 + BPH17-ptb*-PYL (84.0%), showed the highest ADM rates among PYLs with two genes and were higher than any of the corresponding NILs. The ADM rates of seven PYLs—*BPH3* + *BPH17*-PYL, *BPH17* + *BPH21*-PYL, *BPH20* + *BPH32*-PYL, *BPH21* + *BPH25*-PYL, *BPH21* + *BPH17-ptb*-PYL, *BPH25* + *BPH17-ptb*-PYL and *BPH32* + *BPH17-ptb*-PYL, ranged from 50.0% to 68.0%, while the ADM of *BPH20* + *BPH21*-PYL was 33.3%. The ADM rates of PYLs for three genes—*BPH2 + BPH3 + BPH17*-PYL (96.0%), *BPH2 + BPH32 + BPH17-ptb*-PYL (95.8%) and *BPH20 + BPH21 + BPH32*-PYL (92.0%), were higher than those of the corresponding NILs and PYLs for two genes, and were similar to the ADM rates of the donor parents (100%). Furthermore, the ADM rates were negatively correlated with the DSs (Pearson’s C = −0.79; *p* < 0.001) and FBRRs (Pearson’s C = −0.76; *p* < 0.001).

### 2.5. Comparison of ADM Rates for the Koshi-2013 Population on the NILs and PYLs

T65 was susceptible to the Koshi-2013 population with an ADM of 5.0%. Rathu Heenati had the highest ADM among entries (84.0%), which was significantly higher than T65. The ADM rates of the other donor parents, IR71033-121-15 (44.0%) and PTB33 (36.0%), were lower than that of Rathu Heenati. The ADM rates of the NILs were less than or equal to 20.0%. Among the PYLs, the ADM rates of *BPH2* + *BPH17*-PYL (32.0%), *BPH20* + *BPH32*-PYL (36.0%) and *BPH2* + *BPH3* + *BPH17*-PYL (36.0%) were highest. The ADM rates of the six PYLs, *BPH3* + *BPH17*-PYL, *BPH20* + *BPH21*-PYL, *BPH21* + *BPH25*-PYL, *BPH32* + *BPH17-ptb*-PYL, *BPH2* + *BPH32* + *BPH17-ptb*-PYL and *BPH20* + *BPH21* + *BPH32*-PYL ranged from 20% to 28%. The ADM rates of the other PYLs, *BPH2* + *BPH32*-PYL, *BPH2* + *BPH17-ptb*-PYL, *BPH17* + *BPH21*-PYL, *BPH25* + *BPH17-ptb*-PYL, *BPH2* + *BPH25*-PYL and *BPH21* + *BPH17-ptb*-PYL ranged from 8.0% to 16.0%.

### 2.6. Agronomic Characteristics of the NILs and PYLs

Six agronomic traits—days to heading (DTH), panicle length (PL), culm length (CL), flag leaf length (LL), flag leaf width (LW) and panicle number per plant (PN) of the NILs and PYLs are presented in Table 5. The DTHs and PNs of the NILs and PYLs were not significantly different from those of T65. The PLs, CLs, LLs and LWs were similar for NILs and T65, except that the *BPH2*-NIL had longer culms, *BPH25*-NIL had shorter panicles and *BPH3*-NIL had wider flag leaves. The PLs, CLs, LLs and LWs were not significantly different between the PYLs and T65, except that *BPH2 + BPH17*-PYL, *BPH2 + BPH25*-PYL, *BPH2 + BPH32*-PYL, *BPH2 + BPH17-ptb*-PYL, *BPH21 + BPH25*-PYL and *BPH2 + BPH3 + BPH17*-PYL had longer culms; *BPH21 + BPH25*-PYL had longer flag leaves; *BPH2 + BPH32*-PYL, *BPH17 + BPH21*-PYL and *BPH20 + BPH32*-PYL had narrower flag leaves; and *BPH3 + BPH17*-PYL had wider flag leaves. 

## 3. Discussion

The seven NILs we developed carried BPH resistance genes on the short arm of chromosome 4 (*BPH17*-NIL, *BPH20*-NIL and *BPH17-ptb*-NIL), on the short arm of chromosome 6 (*BPH3*-NIL and *BPH32*-NIL) and on the long arm of chromosome 12 (*BPH2*-NIL and *BPH21*-NIL). One of the resistance genes on chromosome 12, *BPH2*, was originally identified from ASD7 which was used as a donor parent for many modern resistant varieties (e.g., IR36, IR42 and so on) [31,67]. *BPH2* from ASD7 is identical to *BPH26* in DNA sequence and resistance level [10]. *BPH2* from ASD7 was resistant against the Hatano-66 population (synonym of Hadano-66) but susceptible to Nishigoshi-05, a BPH population collected in Koshi, Kumamoto Prefecture in 2005 [51]. PTB33 was reported to carry one dominant and one recessive gene [40] that were confirmed to be *BPH2* and *BPH3* using conventional genetic analysis [41]. However, there was no report of the exact location of *BPH2* from PTB33. In our study, *BPH2*-NIL had similar resistance patterns to *BPH2* in ASD7: *BPH2*-NIL was highly resistant (ADM of 68.9%) against the Hadano-66 population but less effective (ADM of 4.0%) against the recently collected population, Koshi-2013. Moreover, *BPH2*-NIL (PTB33) and *BPH26*-NIL had similar resistance levels against both Hadano-66 and Koshi-2013, suggesting that PTB33, ADR52 and ASD7 might harbor the same resistance gene. Further sequence analysis for *BPH2* from PTB33 is necessary to understand its genetic basis. Another gene on chromosome 12, *BPH21,* was originally identified from IR71033-121-15, an introgression line derived from *O. minuta* and estimated to be located between two markers, S12094A and B122, on the long arm of chromosome 12 [17]. Recently, *BPH21* has been reported to be allelic to *BPH26* [6] and *BPH18* [68] based on amino acid sequences. Both *BPH18* and *BPH26* were isolated and located at 22.9 Mbp on chromosome 12 [9,10]. Therefore, we estimated that the location of *BPH21* was around 22.9 Mbp on chromosome 12, and the region carrying *BPH21* from IR71033-121-15 was selected using RM1246 (19.2 Mbp) and RM28493 (23.3 Mbp) in this study.

The *BPH17* locus on chromosome 4S from Rathu Heenati has been reported by Sun et al. (2005) [16]. *BPH17* was mapped between two markers, RHD9 (6.2 Mbp) and RHC10 (7.0 Mbp), on chromosome 4S and isolated by Liu et al. (2014) [8]. The amino acid sequence and chromosomal location of *BPH17* from Rathu Heenati were the same as those of *BPH17-ptb* from PTB33 [8]. In this study, resistance of *BPH17*-NIL and *BPH17-ptb*-NIL against the Hadano-66 population differed; however, both NILs had similar effects on the Koshi-2013 population. The different resistant levels might be because the loci were derived from different accessions or varieties of rice. Therefore, the amino acid sequences of PTB33 and Rathu Heenati used in this study on the *BPH17* locus should be determined for future research. Additionally, *BPH20* was detected between two markers, B42 (8.7 Mbp) and B44 (8.9 Mbp) on chromosome 4 [17]. Two NILs for *BPH17* and *BPH20* on the genetic background of 9311 varieties developed by Xiao et al (2016) [51] showed different resistance levels against a BPH population from China [68]. In our study, the resistance levels of *BPH17* and *BPH20* were different in both MSST and antibiosis tests against the Hadano-66 population and against the Koshi-2013 population, which corresponds well with previous research by Xiao et al. (2016) [51]. Therefore, the genes on chromosome 4S of IR71033-121-15, PTB33 and Rathu Heenati might be different. To confirm this, further sequence analyses are needed for the three loci *BPH17, BPH17-ptb* and *BPH20*.

Among six genes/QTLs that have been identified on the short arm of chromosome 6 of *O. sativa* and its wild relatives [3,12], *BPH3* and *BPH32* have been widely introduced to elite rice cultivars to improve BPH resistance and were related to durable and broad-spectrum resistance in PTB33 and Rathu Heenati [67,69]. In previous research, *BPH3* was mapped onto chromosome 6 between two markers, RM19291 (1.2 Mbp) and RM8072 (1.4 Mbp) [69]. *BPH32* from PTB33 was identified at the same location as *BPH3* from Rathu Heenati, but the amino acid sequence of *BPH3* was not identical to that of *BPH32* [12]. In our study, the resistance levels of the *BPH3*-NIL were slightly different from those of the *BPH32*-NIL, suggesting that *BPH3* might be different from *BPH32*. A comparison of amino acid sequences between *BPH3* and *BPH32* would be necessary to confirm whether these resistance genes are different.

Among the developed NILs, the *BPH3*-NIL, *BPH17*-NIL, *BPH17-ptb*-NIL and *BPH32*-NIL had around 97.0% of their chromosomal segments from the recurrent parent. This proportion coincides with the theoretical ratio for substituting chromosomal segments from recurrent parents by backcrossing four times. The other NILs had fewer chromosomal segments from T65 than the theoretical rate. The substituted chromosomal segments from the donor parents might be related to undesirable traits such as the suppression of the associated BPH resistance gene. Additionally, due to the low density of available polymorphic SSR markers between T65 and donor DNA around the target genes, the BPH resistance genes on the NILs were selected using two flanking markers that were relatively far apart. Furthermore, *BPH17*, *BPH20* and *BPH17-ptb* on the NILs were selected by flanking markers with longer intervals because of the low density of polymorphic markers between donor parents and T65 around the chromosome 4S region. The intervals between each of the flanking marker pairs for *BPH17* and *BPH17-ptb* were 3.7 Mbp, and that for *BPH20* was 5.7 Mbp. Similarly, the interval for each of the two flanking markers for *BPH2* and *BPH21* on chromosome 12 was 4.1 Mbp because the exact locations of genes had not been identified before we started to develop the NILs by MAS. Therefore, many of the NILs had relatively long chromosome segments derived from the donor parents and there is a possibility that the remaining chromosomal segments from donor DNA around the target genes included linkage drag associated with susceptibility. In further research, ensuring that flanking markers are tightly linked to target genes will avoid linkage drag from donors through MAS and backcrossing.

An improvement of rice resistance levels against BPH is necessary since many genes have become less effective against BPH across Asia [45]. In this study, we developed 15 PYLs carrying two or three genes for BPH resistance. The PYLs tended to increase resistance against the two BPH populations, Hadano-66 and Koshi-2013. Among the 15 PYLs, 12 and nine PYLs had higher ADM rates than corresponding NILs against Hadano-66 and Koshi-2013, respectively; ten PYLs had lower FBRRs compared to corresponding NILs in the MSST against the Hadano-66 population. For example, *BPH2 + BPH32*-PYL (84.0%) and *BPH2 + BPH32 + BPH17-ptb*-PYL (95.8%) had higher resistance levels than those of the *BPH2*-NIL (68.9%), *BPH32*-PYL (14.0%) and *BPH17-ptb*-PYL (22.0%) in antibiosis tests against the Hadano-66 population. The ADM rates of *BPH2 + BPH17*-PYL (32.0%) and *BPH2 + BPH3 + BPH17*-PYL (36.0%) were higher than those of *BPH2*-NIL (4.0%), *BPH3*-NIL (0%) and *BPH17*-NIL (20.0%) against the Koshi-2013 population. Additionally, the FBRR of *BPH2 + BPH17* (42.3%) was lower than for *BPH2*-NIL (67.6%) and *BPH17*-NIL (58.8%). However, the effectiveness of the PYLs was not consistently higher than that of the corresponding NILs. The effect of PYLs was influenced by specific interactions between gene loci, the specific BPH populations and the screening methods. For example, the resistance levels of *BPH3 + BPH17*-PYL (50.0%) and *BPH17 + BPH21*-PYL (58.3%) were not higher than that of *BPH17*-NIL (59.0%) in antibiosis tests (or ADM rates) against the Hadano-66 population. *BPH2 + BPH25*-PYL (87.5%) showed higher ADM against the Hadano-66 population in comparison to *BPH2*-NIL (68.9%) and *BPH25*-NIL (16.0%), while the ADM rate of *BPH2 + BPH25*-PYL (12.0%) was lower than that of *BPH25*-NIL (16.0%) against the Koshi-2013 population. A similar tendency has been reported for gene combinations between *BPH1* and *BPH2* [70]; *BPH18* and *BPH32*; *BPH20* and *BPH32*; and *BPH2, BPH18* and *BPH32* [57]. That the resistance levels of most of the PYLs were not significantly higher than those of the corresponding NILs might be related to the relatively small number of replications used in the bioassays (five replications for the antibiosis and three replications for the MSST).

In a previous study, virulence of a BPH population collected during 2005 in Japan had increased compared with the virulence of a population collected in 1966 [51]. Through antibiosis tests, we evaluated BPH resistance against the populations collected in 1966 (Hadano-66) and in 2013 (Koshi-2013). Both represented BPH arriving as migrants to Japan. The Hadano-66 population was virulent to T65 (with no resistance gene) but avirulent to all plants with resistance genes, including Mudgo (*BPH1*), ASD7 (*BPH2*), Rathu Heenai (*BPH3* and *BPH17*), Babawee (*BPH4*), Chin Saba (*BPH8*), Balamawee (*BPH9*) and two NILs, *BPH25*-NIL and *BPH26*-NIL [51,71]. In the present study, most of the NILs, all of the PYLs and the donor parents were still effective against the Hadano-66 population. In contrast, all of the NILs and most of the PYLs were susceptible to the Koshi-2013 population, suggesting that BPH recently arriving to Japan from China has greater virulence than was evident about 50 years ago (i.e., 1966). Among the PYLs, two PYLs, *BPH20 + BPH32*-PYL and *BPH2 + BPH3 + BPH17*-PYL, had relatively high resistance, suggesting that PYLs with combinations of these genes are likely to provide good resistance against the current BPH populations that arrive to Japan (Koshi-2013). Finding new sources of resistance genes will be necessary to further improve resistance against contemporary BPH populations as they gain virulence.

In comparison to the corresponding NILs and PYLs, the resistance levels of PTB33, Rathu Heenati and IR71033-121-15 were higher. This suggests that PTB33, Rathu Heenati and IR71033-121-15 might also contain other BPH resistance gene(s). The other genetic factor(s) for BPH resistance can be revealed by analyzing the segregating populations derived from crosses between the developed PYLs and their donor parents in future studies. Additionally, Rathu Heenati had *QBPH4.1* (5.8-7.8 Mbp) and *QBPH4.2* (15.2-17.2 Mbp) on chromosome 4S rather than *BPH3* and *BPH17* [13]. Therefore, the NILs and PYLs carrying *QBPH4.1* and *QBPH4*.2 should be developed and evaluated in further analyses. On the other hand, the lower resistance levels of the NILs and PYLs might be related to the relatively high ratio of substituted chromosomal segments from donors in the NILs (from 3.8 to 55.0 Mbp) and PYLs. There is a possibility that the retained donor chromosomal segments in the genetic background of the NILs and PYLs might be linked to the suppression of BPH resistance. To gain further knowledge of BPH resistance controlled by multiple genes, it will be essential to reduce the donor parent chromosomal segments on the NILs and PYLs by further backcrossing and MAS.

## 4. Materials and Methods

### 4.1. Plant Materials

**Figure 3:**
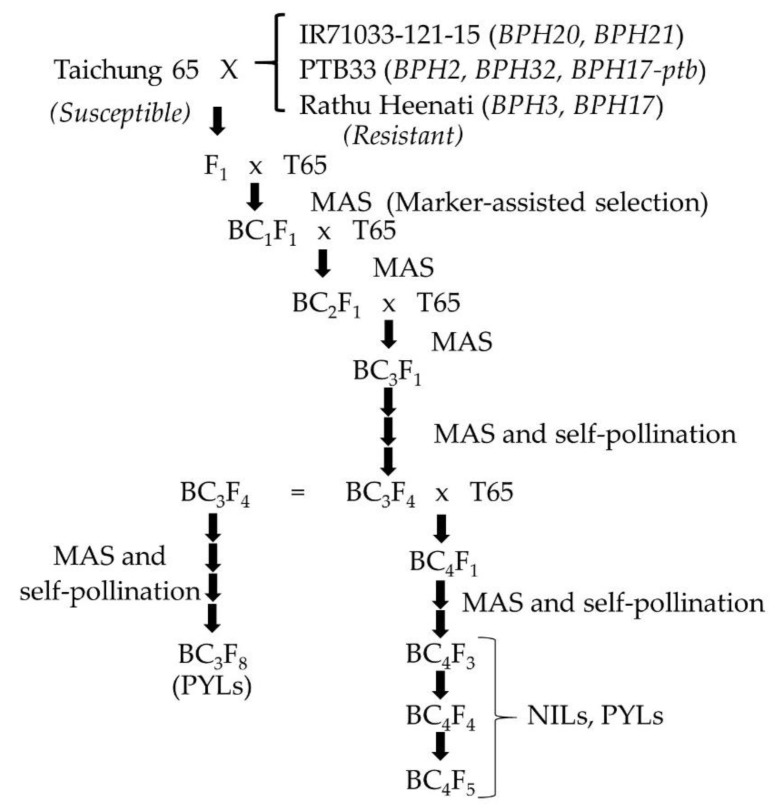
Breeding scheme for the development of near-isogenic lines and pyramided lines containing brown planthopper resistance genes from donor parents, IR71033-121-15, PTB33 and Rathu Heenati.

To develop NILs with BPH resistance genes, a *japonica* rice variety, T65, that is susceptible to BPH, was used as a recurrent parent, and three rice varieties resistant to BPH were donor parents. The donor lines were IR71033-121-15, PTB33 and Rathu Heenati. IR71033-121-15 contains two BPH resistance genes, *BPH20* and *BPH21*, from the wild rice species *O. minuta* (Accession number: IRGC101141) [17]. PTB33 (Accession number: IRGC19325) that originated from India contains *BPH2, BPH17-ptb* and *BPH32.* Rathu Heenati (Acc. no. IRGC 11730), that originated from Sri Lanka, carries *BPH3* and *BPH17* [16,39]. T65 was crossed with these donor parents and F_1_ plants were backcrossed four times with T65 to generate BC_4_F_1_ plants (Figure 3). At each generation of backcrossing, plants carrying BPH resistance genes from the donor parents were selected by MAS using flanking SSR markers of the target BPH resistance genes (Table 1). The selected BC_4_F_1_ plants were self-pollinated to produce BC_4_F_3_, BC_4_F_4_ and BC_4_F_5_ plants with BPH resistance genes. Finally, seven NILs with either *BPH2, BPH3, BPH17, BPH20, BPH21, BPH32* or *BPH17-ptb* were developed. The NILs were used to survey the genetic background and evaluate BPH resistance levels as well as agronomic traits. Two additional NILs, *BPH25*-NIL and *BPH26*-NIL were used in the development of the PYLs [54]. 

### 4.2. The Development of PYLs with BPH Resistance Genes

All the PYLs for two or three BPH resistance genes were developed using the NILs descended from the BC_4_F_1_ generation, except *BPH20* + *BPH21*-PYL and *BPH32* + *BPH17-ptb*-PYL that were descended from the BC_3_F_1_ generation. The F_1_ plants derived from crosses between NILs were self-pollinated to produce F_2_ plants. From 96 F_2_ plants, plants that were homozygous for two or three BPH resistance genes were selected by MAS. Several plants from 96 F_3_ plants with similar agronomic traits to T65 were selected as final PYLs. The following PYLs carrying two or three BPH resistance genes were evaluated for BPH resistance and agronomic traits: *BPH2 + BPH17*-PYL, *BPH2 + BPH25*-PYL, *BPH2 + BPH32*-PYL, *BPH2 + BPH17-ptb*-PYL, *BPH3 + BPH17*-PYL, *BPH17 + BPH21*-PYL, *BPH20 + BPH21*-PYL, *BPH20 + BPH32*-PYL, *BPH21 + BPH25*-PYL, *BPH21 + BPH17-ptb*-PYL, *BPH25 + BPH17-ptb*-PYL, *BPH32 + BPH17-ptb*-PYL, *BPH2 + BPH3 + BPH17*-PYL, *BPH2 + BPH32 + BPH17-ptb*-PYL and *BPH20 + BPH21 + BPH32*-PYL.

### 4.3. The MAS for BPH Resistance Genes

To conduct MAS, approximately 2 cm of leaves from two–week old seedlings were collected and dried in a freeze drier for 48 h, and total DNA was extracted using the potassium acetate method [72]. The genotypes of SSR markers on plants in each generation were determined by polymerase chain reaction (PCR) and electrophoresis. The PCR amplification mix (8 µL) contained 3 μL of 1X GoTaq^®^ Green Master Mix (pH 8.5), 0.25 μM of primer and 4 μL of 20 times-diluted DNA. Each PCR amplification included one cycle at 96 °C for 5 min, 35 cycles at 96 °C for 30 s, 55 °C for 30 s and 72 °C for 30 s, followed by one extension cycle at 25 °C for 1 min. PCR products were analyzed by electrophoresis at 200 V using 4% agarose gel with 0.5 μg/mL ethidium bromide in 0.5X TBE buffer for 1 h and photographed under ultraviolet light. During MAS for resistance genes on chromosome 4S, the plants with *BPH17* and *BPH17-ptb*, were selected using two markers, RM8213 and MS10, and the plants with *BPH20* were selected using MS10 and RM5900 (Table 1). The plants with *BPH3* and *BPH32* on the short arm of chromosome 6 were selected using two flanking markers, RM508 and RM588. The plants carrying *BPH2* and *BPH21* located on the long arm of chromosome 12 were screened using RM1246 and RM28493. The plants with *BPH25* were selected using S00310 and MSSR1, and the plants with *BPH26* were selected using RM309, RM28438, InD14, RM28466, RM28481 and MSSR2. 

### 4.4. The Genetic Background Survey of the NILs

In the genetic background survey of the NILs, the bulk DNA from five plants was used. A total of 384 SSR markers distributed on 12 rice chromosomes were used during polymorphism tests with T65 and the donor parents [62]. Among the 384 SSR markers, 254 SSR markers with polymorphisms between IR71033-121-15 and T65 were utilized to identify substituted chromosomal segments from IR71033-121-15 on *BPH20*-NIL and *BPH21*-NIL. Additionally, 244 of 384 SSR markers with polymorphisms between PTB33 and T65 were used to detect substituted chromosomal segments from PTB33 on *BPH2*-NIL, *BPH32*-NIL and *BPH17-ptb*-NIL. To identify substituted chromosomal segments from Rathu Heenati on *BPH3*-NIL and *BPH17*-NIL, 204 of 384 SSR markers with polymorphisms between Rathu Heenati and T65 were used. The whole genome compositions of the developed NILs were graphically displayed following the concept of the graphical genotype proposed by Young and Tanksley (1989) using GGT software version 2.0 [73].

### 4.5. The BPH Populations and the Characterization of BPH Resistance

Two BPH populations from Japan (Hadano-66 and Koshi-2013) were used to evaluate the NILs and PYLs for their resistance. Hadano-66 was collected in Hadano City, Kanagawa Prefecture, Japan in 1966 [51], and Koshi-2013 was collected in Koshi City, Kumamoto Prefecture, Japan in 2013. Both BPH strains were maintained on the susceptible *japonica* rice variety, Reiho, at 25 °C with 16 h/8 h of light/dark at Kyushu Okinawa Agricultural Research Center of the National Agriculture and Food Research Organization in Japan. 

To evaluate resistance, an adaptation of the modified seedbox screening test (MSST) [45,74] was applied at 25 °C using the Hadano-66 strain. To conduct the test, 30 seeds of each of the NILs, PYLs and parent lines were sown to single rows in a plastic tray (23.0 × 30.0 × 2.5 cm) with 2.5 cm between successive rows of seedlings. Two sets of trays—one tray infested by BPH and the other without infestation (the control tray), were used to measure the effects of BPH on plant biomass. One row of Rathu Heenati was added as a resistant control, while three rows of T65 were sown at the center and the two edges as a susceptible control. At seven days after sowing (DAS), the plants in the trays were thinned to 20 plants per row. One tray was infested by the second and third instar nymphs at a density of around 20 BPHs per plant. The experiment was replicated three times. When all the plants of T65 were completely desiccated due to BPH feeding, the DSs of all lines were graded following the standard evaluation system for rice of the International Rice Research Institute [75]. The plants from each row in the two trays were cut above the soil surface and weighed. The fresh biomass reduction rate (FBRR) was calculated using the following formula:

(1) $$\begin{array}{c} \mathrm{Fresh}\;\mathrm{biomass}\;\mathrm{reduction}\;\mathrm{rate}\\ \left(\mathrm{FBRR}\right)\left(\%\right) \end{array}=\left[1-\frac{\mathrm{Infested}\;\mathrm{plant}\;\mathrm{weight}\;(g)}{\mathrm{Non-infested}\;\mathrm{plant}\;\mathrm{weight}\;(g)}\right]\times 100.$$

### 4.6. Antibiosis Tests

Antibiosis tests were conducted at 25 °C following the method described by Myint et al. (2009) [51]. Five plants of each NIL, PYL and parent line were individually sown in 200 mL plastic cups. At four weeks after sowing, the plants were trimmed to 15 cm height and covered with a plastic cage with insect screen windows for ventilation. Each cage was infested with five thin-abdomen brachypterous female BPHs. At five days after infestation, the ADM was recorded (i.e., the number of dead females).

### 4.7. Characterization of NILs and PYLs for Agronomic Traits

The NILs and PYLs were grown in a paddy field at Saga University (Saga, Japan) in 2018 and characterized for their agronomic traits compared to those of T65. Seedlings were transplanted at 28 DAS as one plant per hill, with 20 cm between hills and 25 cm between rows. Each entry was planted as at least three rows (12 plants per a row). Six agronomic traits: DTH, CL, PL, LL, LW and PN were measured for five plants in the same row. DTH was the days from sowing until 50% of panicles flowered. CL was measured from the soil surface to the panicle neck. PL is the length from tip to panicle neck of the longest panicle. The flag leaf width and length were measured from the largest and longest flag leaf of each sampled plant. Panicle number is the number of reproductive panicles of each plant at maturity.

### 4.8. Statistical Analysis

Mean values of BPH resistance (DS, FBRR and ADM) for the NILs and PYLs and agronomic traits were compared using one-way ANOVA. Dunnett’s test and Tukey Kramer’s test were conducted for multiple comparisons of BPH resistance and agronomic traits, respectively, using R software version 3.5.2.

## Figures and Tables

**Table 1:** The SSR markers used for maker-assisted selection of nine genes for resistance to the brown planthopper.

Marker	Resistance Gene Tagged	Chromosome	Forward Primer Sequence (5′→3′)	Reverse Primer Sequence (5′→3′)	Physical Position (Mbp) *	Predicted Size (bp) **
RM1246 ^a^	*BPH2, BPH21*	12	GGCTCACCTCGTTCTCGATCC	CATAAATAAATAGGGCGCCACACC	19.16	195
RM28493 ^b^	*BPH2, BPH21*	12	ACCGTTAGATGACACAAGCAACG	GGTTAGCAAGACTGGAGGAGACG	23.28	259
RM508 ^c^	*BPH3, BPH32*	6	AGAAGCCGGTTCATAGTTCATGC	ACCCGTGAACCACAAAGAACG	0.44	158
RM588 ^c^	*BPH3, BPH32*	6	TCTTGCTGTGCTGTTAGTGTACG	GCAGGACATAAATACTAGGCATGG	1.61	97
RM8213 ^a^	*BPH17, BPH17-ptb*	4	TGTTGGGTGGGTAAAGTAGATGC	CCCAGTGATACAAAGATGAGTTGG	4.42	178
MS10 ^d^	*BPH17, BPH17-ptb, BPH20*	4	CAATACGAGAAGCCCCTCAC	CTGAAGGAACACGCGGTAGT	8.08	167
RM5900 ^a^	*BPH20*	4	TTCTACGTTTGACCGTCA	TCTAGGAGCGTTTGTAGGAG	13.77	248
S00310 ^e^	*BPH25*	6	CAACAAGATGGACGGCAAGG	TTGGAAGAAAAGGCAGGCAC	0.21	215
MSSR1 ^e^	*BPH25*	6	CTAGCTGCTCTGCTCTGCTG	CGGCAATCTCTCCGAATC	0.22	114
RM309 ^f^	*BPH26*	12	CACGCACCTTTCTGGCTTTCAGC	AGCAACCTCCGACGGGAGAAGG	21.52	177
RM28438 ^b^	*BPH26*	12	GTTCGTGAGCCACAACAAATCC	GTTAAATGCTCCACCAAACACACC	22.59	216
InD14 ^g^	*BPH26*	12	GGCCGAGTAGGATACTCTAGAAA	CTGCGAGAAAGGAGAGGTGG	22.87	387
RM28466 ^b^	*BPH26*	12	CCGACGAAGAAGACGAGGAGTAGCC	AGGCCGGAGAGCAATCATGTCG	22.98	93
RM28481 ^b^	*BPH26*	12	GTCAATTAACCATTGCCCATGC	TTCACGTGGGAACTACTCATGC	23.14	242
MSSR2 ^e^	*BPH26*	12	CATGTCGAAGAGGTTGCAGA	GGTTTCATCCAAGTCCACGA	25.03	265

Primer sequence information was obtained from: ^a^: McCouch et al. (2002) [62], ^b^: International Rice Genome Sequencing Project (IRGSP) (2005) [63], ^c^: Temnykh et al. (2001) [64], ^d^: Rahman et al. (2009) [17], ^e^: Myint et al. (2012) [29], ^f^: Temnykh et al. (2000) [65] and ^g^: Zhao et al. (2016) [6]. * The physical position of marker was the physical location of forward primer for each marker obtained from The Rice Annotation Project Database [66]. ** Physical distance and predicted size were estimated on the basis of the Nipponbare genome sequence.

**Table 2:** Details of the seven near-isogenic lines and 15 pyramided lines carrying brown planthopper resistance genes.

Entry	Gene (Donor Parent)						Generation
***BPH2-NIL***	***BPH2***	**(PTB33)**					BC_4_F_3_
*BPH3-NIL*	*BPH3*	(Rathu Heenati)					BC_4_F_4_
*BPH17-NIL*	*BPH17*	(Rathu Heenati)					BC_4_F_4_
*BPH20-NIL*	*BPH20*	(IR71033-121-15)					BC_4_F_5_
*BPH21-NIL*	*BPH21*	(IR71033-121-15)					BC_4_F_5_
*BPH32-NIL*	*BPH32*	(PTB33)					BC_4_F_4_
*BPH17-ptb-NIL*	*BPH17-ptb*	(PTB33)					BC_4_F_3_
*BPH2+BPH17-PYL*	*BPH2*	(PTB33)	*BPH17*	(Rathu Heenati)			BC_4_F_3_ equivalent
*BPH2+BPH25-PYL*	*BPH2*	(PTB33)	*BPH25*	(ADR52)			BC_4_F_3_ equivalent
*BPH2+BPH32-PYL*	*BPH2*	(PTB33)	*BPH32*	(PTB33)			BC_4_F_3_ equivalent
*BPH2+BPH17-ptb-PYL*	*BPH2*	(PTB33)	*BPH17-ptb*	(PTB33)			BC_4_F_3_ equivalent
*BPH3+BPH17-PYL*	*BPH3*	(Rathu Heenati)	*BPH17*	(Rathu Heenati)			BC_4_F_4_ equivalent
*BPH17+BPH21-PYL*	*BPH17*	(Rathu Heenati)	*BPH21*	(IR71033-121-15)			BC_4_F_3_ equivalent
*BPH20+BPH21-PYL*	*BPH20*	(IR71033-121-15)	*BPH21*	(IR71033-121-15)			BC_3_F_8_ equivalent
*BPH20+BPH32-PYL*	*BPH20*	(IR71033-121-15)	*BPH32*	(PTB33)			BC_4_F_3_ equivalent
*BPH21+BPH25-PYL*	*BPH21*	(IR71033-121-15)	*BPH25*	(ADR52)			BC_4_F_3_ equivalent
*BPH21+BPH17-ptb-PYL*	*BPH21*	(IR71033-121-15)	*BPH17-ptb*	(PTB33)			BC_4_F_3_ equivalent
*BPH25+BPH17-ptb-PYL*	*BPH25*	(ADR52)	*BPH17-ptb*	(PTB33)			BC_4_F_3_ equivalent
*BPH32+BPH17-ptb-PYL*	*BPH32*	(PTB33)	*BPH17-ptb*	(PTB33)			BC_3_F_8_ equivalent
*BPH2+BPH3+BPH17-PYL*	*BPH2*	(PTB33)	*BPH3*	(Rathu Heenati)	*BPH17*	(Rathu Heenati)	BC_4_F_3_ equivalent
*BPH2+BPH32+BPH17-ptb-PYL*	*BPH2*	(PTB33)	*BPH32*	(PTB33)	*BPH17-ptb*	(PTB33)	BC_4_F_3_ equivalent
*BPH20+BPH21+BPH32-PYL*	*BPH20*	(IR71033-121-15)	*BPH21*	(IR71033-121-15)	*BPH32*	(PTB33)	BC_4_F_3_ equivalent

**Table 3:** Background survey analysis of seven near-isogenic lines using SSR polymorphic markers.

NIL	Donor	No. of SSR Markers			Genome Ratio (%)		Total Physical Distance of Donor Segment (Mbp) *	
		T65	Donor	Total	T65	Donor		
*bph2*-NIL	PTB33	183	20	203	85.2–90.9	9.1–14.8	33.9	55.0
*Bph3*-NIL	Rathu Heenati	181	14	195	97.0–99.0	1.0–3.0	3.8	11.3
*Bph17*-NIL	Rathu Heenati	170	3	173	95.2–99.0	1.0–4.8	3.8	17.6
*BPH17-ptb*-NIL	PTB33	219	10	229	92.4–97.2	2.8–7.6	10.5	28.1
*Bph20*-NIL	IR71033-121-15	224	13	237	90.4–94.4	5.6–9.6	20.6	35.5
*Bph21*-NIL	IR71033-121-15	210	19	229	88.4–92.9	7.1–11.6	26.4	43.1
*BPH32*-NIL	PTB33	220	13	233	95.9–98.1	1.9–4.1	7.1	15.1

* The minimum physical distance of donor segment was calculated by the distance between two markers delimiting the substituted segment and the maximum amount was calculated by two flanking markers of substituted segments.

**Table 4:** The adult mortality of *Nilaparvata lugens* on near-isogenic lines and pyramided lines carrying brown planthopper resistance genes.

Entry	Adult Mortality (%)	
	Hadano-66	Koshi-2013
*BPH2-NIL*	68.9 ± 28.5 ^abcd^	4.0 ± 8.9 ^b^
*BPH3*-NIL	30.0 ± 38.0 ^def^	0.0 ± 0.0 ^b^
*BPH17-NIL*	59.0 ± 25.1 ^abcde^	20.0 ± 14.1 ^b^
*BPH20-NIL*	24.0 ± 22.7 ^def^	4.0 ± 8.9 ^b^
*BPH21-NIL*	36.0 ± 37.5 ^cdef^	12.0 ± 17.9 ^b^
*BPH25-NIL*	16.0 ± 15.8 ^f^	16.0 ± 16.7 ^b^
*BPH26-NIL*	50.0 ± 41.4 ^abcdef^	4.0 ± 8.9 ^b^
*BPH32*-NIL	14.0 ± 16.5 ^f^	12.0 ± 17.9 ^b^
*BPH17-ptb-NIL*	22.0 ± 19.9 ^ef^	20.0 ± 20.0 ^b^
*BPH2+BPH17-PYL*	75.0 ± 19.5 ^abcd^	32.0 ± 17.9 ^b^
*BPH2+BPH25-PYL*	87.5 ± 17.9 ^ab^	12.0 ± 11.0 ^b^
*BPH2+BPH32-PYL*	84.0 ± 16.7 ^abc^	16.0 ± 16.7 ^b^
*BPH2+BPH17-ptb-PYL*	84.0 ± 16.7 ^abc^	16.0 ± 16.7 ^b^
*BPH3+BPH17-PYL*	50.0 ± 32.5 ^abcdef^	24.0 ± 16.7 ^b^
*BPH17+BPH21-PYL*	58.3 ± 27.5 ^abcdef^	16.0 ± 21.9 ^b^
*BPH20+BPH21-PYL*	33.3 ± 22.2 ^cdef^	24.0 ± 16.7 ^b^
*BPH20+BPH32-PYL*	62.5 ± 38.5 ^abcde^	36.0 ± 21.9 ^b^
*BPH21+BPH25-PYL*	64.0 ± 26.1 ^abcde^	24.0 ± 16.7 ^b^
*BPH21+BPH17-ptb-PYL*	54.2 ± 35.0 ^abcdef^	8.0 ± 17.9 ^b^
*BPH25+BPH17-ptb-PYL*	68.0 ± 26.8 ^abcde^	16.0 ± 16.7 ^b^
*BPH32+BPH17-ptb-PYL*	62.5 ± 32.9 ^abcde^	20.0 ± 14.1 ^b^
*BPH2+BPH3+BPH17-PYL*	96.0 ± 8.9 ^ab^	36.0 ± 38.5 ^b^
*BPH2+BPH32+BPH17-ptb-PYL*	95.8 ± 8.9 ^ab^	20.8 ± 20.1 ^b^
*BPH20+BPH21+BPH32-PYL*	92.0 ± 11.0 ^ab^	28.0 ± 26.8 ^b^
IR71033-121-15	100.0 ± 0.0 ^a^	44.0 ± 16.7 ^ab^
PTB33	100.0 ± 0.0 ^a^	36.0 ± 21.9 ^b^
Rathu Heenati	100.0 ± 0.0 ^a^	84.0 ± 35.8 ^a^
Taichung 65	17.6 ± 16.7 ^f^	5.0 ± 10.0 ^b^

Parameter values (means ± standard deviations) followed by the same letter are not significantly different between each *Nilaparvata lugens* population (*p* < 0.05, Tukey–Kramer multiple comparison tests).

**Table 5:** Agronomic traits of near-isogenic lines and pyramided lines for brown planthopper resistance genes.

Entry	Average of Agronomic Trait (AVE ± SD)					
	DTH (day)	CL (cm)	PL (cm)	LL (cm)	LW (cm)	PN
*BPH2-NIL*	104.0 ± 2.0	120.3 ± 3.2 *	23.8 ± 0.7	36.8 ± 4.5	1.1 ± 0.0	16.6 ± 3.2
*BPH3-NIL*	100.8 ± 1.3	104.3 ± 4.8	18.9 ± 1.0	30.2 ± 3.3	1.3 ± 0.0 *	16.2 ± 1.5
*BPH17-NIL*	98.6 ± 1.3	103.2 ± 7.5	23.4 ± 2.2	33.8 ± 8.0	1.2 ± 0.1	15.2 ± 3.1
*BPH20-NIL*	100.8 ± 1.6	94.2 ± 5.6	20.5 ± 2.0	29.7 ± 4.5	1.1 ± 0.0	14.8 ± 4.1
*BPH21-NIL*	100.8 ± 1.5	104.2 ± 2.9	20.9 ± 1.2	32.6 ± 3.0	1.0 ± 0.1	13.8 ± 2.6
*BPH25-NIL*	100.4 ± 0.9	100.0 ± 1.9	18.1 ± 1.9 **	26.7 ± 2.8	1.1 ± 0.1	17.6 ± 1.1
*BPH26-NIL*	98.4 ± 0.9	102.1 ± 0.9	22.0 ± 1.6	27.3 ± 3.2	1.1 ± 0.0	13.4 ± 1.8
*BPH32-NIL*	100.2 ± 1.8	97.4 ± 1.5	20.9 ± 0.9	29.3 ± 1.0	1.2 ± 0.0	15.2 ± 1.6
*BPH17-ptb-NIL*	99.0 ± 0.0	97.4 ± 2.3	20.7 ± 1.0	29.6 ± 1.9	1.1 ± 0.0	14.6 ± 1.7
*BPH2+BPH17-PYL*	104.6 ± 1.3	115.1 ± 7.9 *	19.8 ± 1.6	27.3 ± 2.6	1.2 ± 0.0	13.8 ± 2.0
*BPH2+BPH25-PYL*	103.0 ± 1.4	116.2 ± 1.7 *	21.0 ± 0.9	30.2 ± 4.7	1.1 ± 0.1	14.2 ± 1.6
*BPH2+BPH32-PYL*	104.8 ± 1.3	111.4 ± 8.0 *	19.6 ± 1.6	27.8 ± 5.5	1.0 ± 0.0 **	17.8 ± 3.7
*BPH2+BPH17-ptb-PYL*	100.8 ± 0.8	110.7 ± 2.3 **	23.0 ± 1.1	26.9 ± 5.0	1.2 ± 0.1	15.2 ± 2.7
*BPH3+BPH17-PYL*	102.4 ± 1.3	103.3 ± 0.5	18.8 ± 0.9	29.7 ± 3.3	1.5 ± 0.1 *	17.4 ± 2.8
*BPH17+BPH21-PYL*	98.0 ± 0.0	91.5 ± 1.8	21.9 ± 1.7	30.4 ± 3.7	1.0 ± 0.1 *	14.6 ± 1.5
*BPH20+BPH21-PYL*	102.8 ± 1.1	77.9 ± 3.3	23.2 ± 2.4	34.7 ± 6.1	1.2 ± 0.0	18.2 ± 2.3
*BPH20+BPH32-PYL*	105.2 ± 0.4	88.3 ± 1.8	19.3 ± 1.7	34.3 ± 5.8	0.9 ± 0.1 *	16.0 ± 5.2
*BPH21+BPH25-PYL*	102.8 ± 1.3	110.5 ± 4.9 **	23.0 ± 1.9	41.7 ± 3.5 *	1.1 ± 0.1	18.0 ± 3.6
*BPH21+BPH17-ptb-PYL*	102.8 ± 2.5	107.5 ± 4.6	22.4 ± 1.6	33.6 ± 4.5	1.1 ± 0.1	16.0 ± 2.6
*BPH25+BPH17-ptb-PYL*	99.6 ± 1.9	99.6 ± 2.4	19.9 ± 2.0	28.9 ± 3.7	1.2 ± 0.1	15.8 ± 4.8
*BPH32+BPH17-ptb-PYL*	99.0 ± 0.0	107.6 ± 3.5	20.4 ± 2.2	26.7 ± 3.4	1.1 ± 0.0	18.0 ± 1.6
*BPH2+BPH3+BPH17-PYL*	103.4 ± 2.9	119.0 ± 2.6 *	22.4 ± 0.7	31.4 ± 2.2	1.1 ± 0.1	16.0 ± 3.4
*BPH2+BPH32+BPH17-ptb-PYL*	100.6 ± 1.1	102.4 ± 4.4	19.1 ± 0.3	26.2 ± 3.2	1.0 ± 0.1	16.6 ± 5.2
*BPH20+BPH21+BPH32-PYL*	101.2 ± 0.4	91.7 ± 3.1	19.3 ± 1.4	26.6 ± 4.5	1.0 ± 0.1	17.8 ± 2.8
Taichung 65	99.2 ± 0.4	91.4 ± 2.7	21.2 ± 0.7	29.7 ± 4.2	1.1 ± 0.1	14.6 ± 1.5

DTH: days to heading, CL: culm length, PL: panicle length, LL: flag leaf length, LW: flag leaf width, PN: panicle number per plant. * *p* < 0.01; ** *p* < 0.05 (Dunnett’s multiple comparison tests against Taichung 65).
